# Be a force for science: an interview with Barbara Schaal and Bill Moran

**DOI:** 10.1093/nsr/nwy141

**Published:** 2018-12-03

**Authors:** Jane Qiu

**Affiliations:** Writes for NSR from Beijing

## Abstract

*The American Association for the Advancement of Science (AAAS), the world's largest general science membership society, is an international non-profit organization with the stated goals of advancing science, engineering and innovation for the benefit of all people, communicating science broadly, defending scientific freedom, providing a voice for science on societal issues, strengthening and diversifying the science and technology workforce, and advancing international cooperation in science. Founded in 1848, AAAS today has individual members from around 100 countries, and is the publisher of the Science family of journals, including the open-access journal Science Advances*.

*NSR talks to Barbara Schaal—an evolutionary biologist at Washington University in St Louis, Missouri, 2017 President of AAAS, former Vice President of the US National Academy of Sciences, and a former advisor of the President's Council of Advisors in Science and Technology under the Obama administration—and also to Bill Moran, the publisher of Science, about why science is a global public good, how basic science is the engine of economic growth and prosperity, the importance of social science, and why the need to defend the free flow of ideas and people across national boundaries is urgent*.

**NSR:** One of the goals of AAAS is to promote science and innovation. What's the best way to go about it?

**Schaal**: The key to providing discoveries and keeping new technologies and new industries growing is to develop policies to foster the entire innovation pipeline—from basic science through the application of research findings. The value of basic research has been called into question repeatedly. This is partly because the connection between free-flowing, curiosity-driven research enterprise and the benefits our societies receive is not straightforward.

It's really difficult for those outside science to see the direct connection—to trace the line between a wide range of seemingly unfocused basic research enterprises and the development of new technologies and industries. The difficulties are mainly twofold. First, it's not possible to predict the utility of a particular research project. One never knows how research in what may appear an arcane area will lead to monumental discoveries that benefit society. Second, it can take a very long time for basic research to prove to be fundamental to a valuable new technology. These two factors—unpredictability of benefits and the time it takes to prove applicability—make basic science easily marginalized.

**NSR:** It's also a long-standing debate in China about how much money should go into basic science and how much to applied research. The country faces a myriad of development challenges. Government officials often demand quick results and have the tendency to focus on applied research to address specific practical issues.

**Schaal:** There would be no applied research without basic science. Basic research takes a long time, years and often decades.

**Figure 1:**
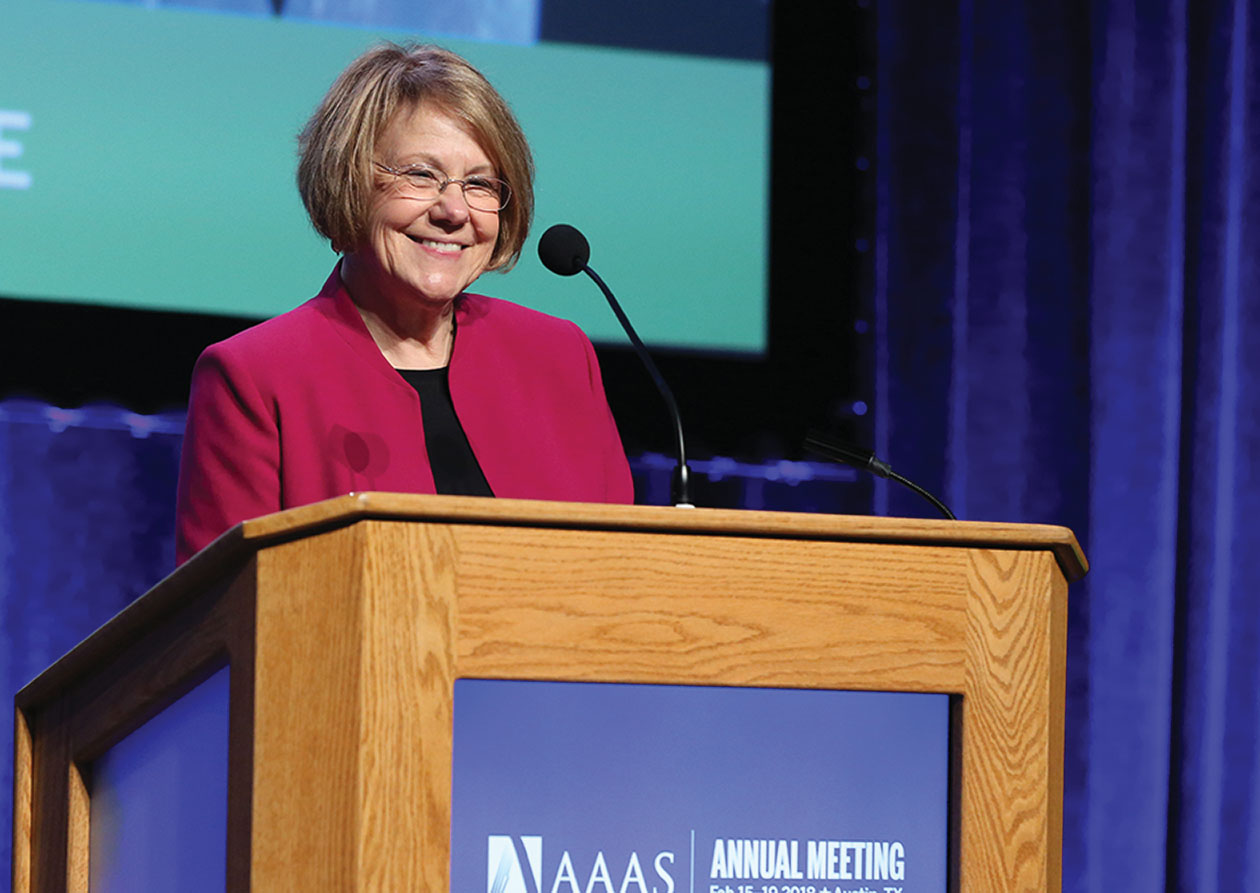
Barbara Schaal, 2017 President of AAAS.

You can’t predict where it is going to go. But it's an investment for the future.

**Moran:** Indeed. Basic research drives innovation. China made the right decision over 20 years ago to invest heavily in basic research. This is why we’ve seen investment to develop schemes like the Thousand Talents Program, which has helped greatly to boost the scientific capability and drive innovation. I’d like to see that continue—not only in China but globally.

**NSR:** How about social science?

**Schaal:** Social science is of great importance. Many challenges that nations face relate to social issues of inequality, disparities in access to healthcare, education, jobs and housing. Social science helps us to understand how we learn, why we behave the way we behave, the role of history in our culture and beliefs, and the role of demographics and immigration in our economies. It has a central role in helping us understand and improve the quality of life for all people. We need policies to support social science—just as we do biological and physical sciences—and we need to try to foster a greater integration between natural and social science.

**NSR:** I came across Chinese scientists who thought that social science was not science.

**Schaal:** That's absolutely wrong. There are enormous data troves on the relationship between race and employment, and there are data troves on immigration and what happens to immigrants. You can ask very crisp clean questions that are of a social nature and get a very hard answer with statistical brackets around it. Such studies are addressing some of the most vexing problems not only for US society but global societies.

Moreover, there is no clear demarcation between science and social sciences in many cases. For instance, anthropology and psychology are often regarded as social-science disciplines. In both fields, you can’t really tell where social anthropology stops and biology begins, and you can’t tell where psychology stops and neuroscience begins. Just as in the sciences, there are extremely powerful methodologies to evaluate data in the social sciences.

**NSR:** What kind of methodologies? Could you give an example?

**Schaal:** Sure. In political sciences, for instance, researchers could do a sort of field experiment. They could explore how changing some of the parameters of an election may cause different voting patterns. This is experimenting with respect to how people in society respond to different factors. It's really remarkable.

The questions social scientists address are not questions about the laws of biology or the laws of physics, but how we work as humans. From a policy perspective, it's extremely important to understand why people behave the way they behave. Equally important is to discern trends. When someone says we are inundated with thousands of criminals from a particular country, for example, we need a way to explore if the actual data back it up.

**NSR:** AAAS has been rather vocal about the free flow of ideas and people. Why is that so important?

**Schaal:** It's important because it's the only way to keep the innovation pipeline viable. Limitations on the ability of scientists to communicate with their peers and the public will harm the scientific enterprise. We need an environment where the lessons of science can be taught without the fear of censure or even in some cases reprisals. We’ve seen pushback in the USA on the teaching of particular science topics, where the subject comes up against political or religious beliefs. This is harming science and the pipeline for a committed workforce, and committed individuals.

Science is not a political construct or a belief system. We are concerned about the weakening of the scientific enterprise, and the long-term harm to the nation and to the global output of science that may result. Open and free communication—communication of ideas, methods and results—is one of the tenets of science. Stifling communication across agencies, across laboratories and countries removes an essential component of the scientific enterprise and ultimately the system will not work.

**Figure 2:**
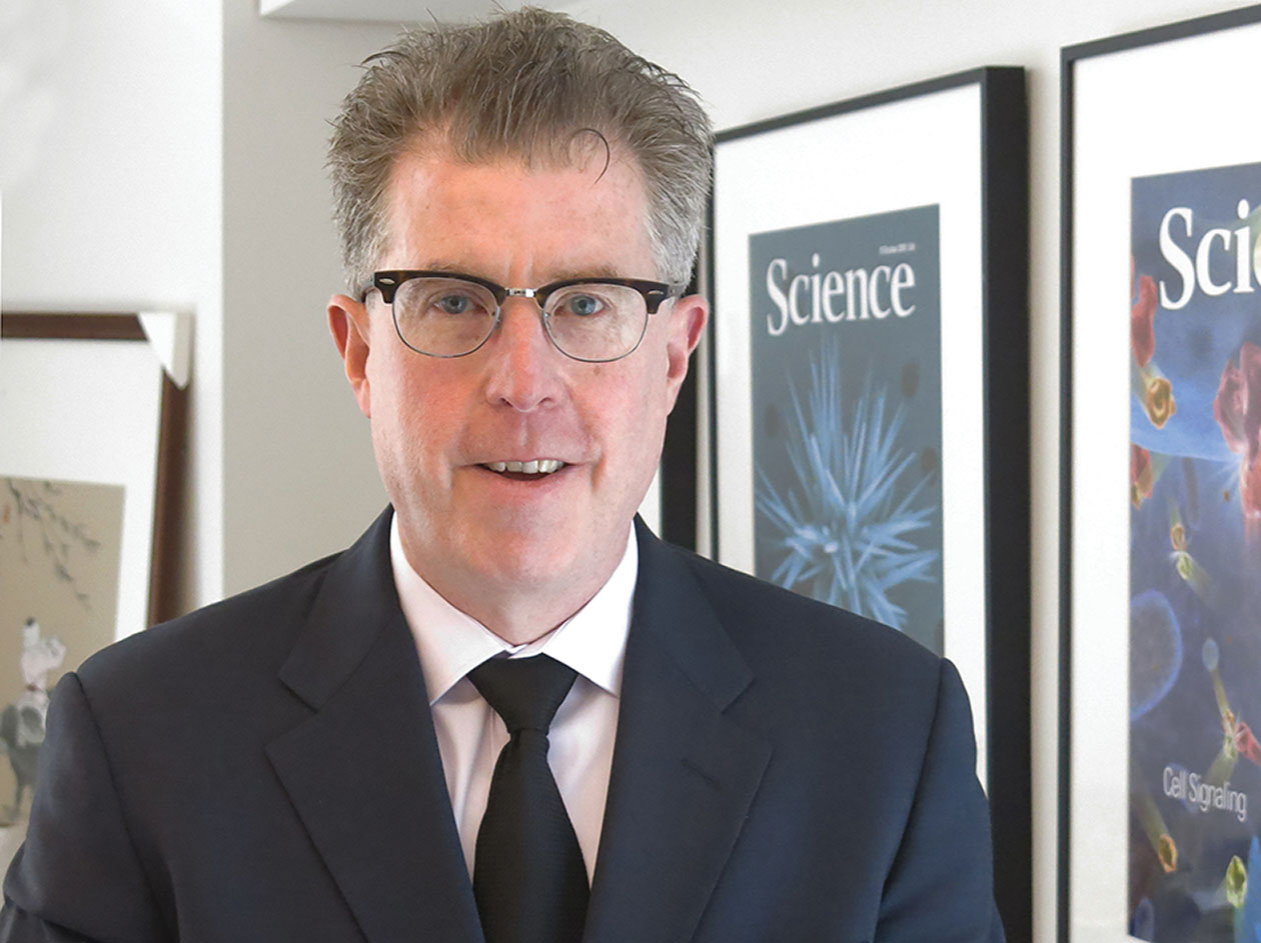
Bill Moran, the publisher of *Science*.

Another concern in the USA is the dampening of the international nature of science, the movement of people across borders. Science is without borders. Just as ideas need to flow across political boundaries, so do people. The USA has benefited probably more than any other country from the international nature of science. We have the great fortune of attracting the best and the brightest from across the world. We have the privilege of educating students from around the world. These students enhance our classrooms and laboratories. Not only do they conduct superb science but they develop friendships and collaborations and, ultimately, promote understanding across borders.

This exchange provides the world and the USA with the benefits of their minds and their work. Policies in the USA and across the globe must keep science international. Government policies must foster the exchange of individuals and ideas, not restrict the exchange of individuals and ideas. Science in the USA flourishes only when it allows the best and the brightest from the world to come and to live and to work here.

**NSR:** I guess that spirit also underlies AAAS’ mission to promote international collaboration. How have you been going about it?

**Schaal:** Not on a one-on-one basis, but really to try to promote global science and the diversity of it, not only among developed countries, but also bringing in developing countries where there usually isn’t a strong tradition of science and technology. That's absolutely important. We know that engaging with people from all around the world, the best, the brightest, the most diverse thinkers from everywhere, enriches the quality of scientific discovery and adds to the global marketplace of ideas.

If you look at most of the exchange, there is tremendous individual collaboration, and that collaboration normally expands because there are joint students, and those students have students. There is a lot of collaboration between individual investigators and individual laboratories. In my university, Washington University in St Louis, we have the MacDonnell International Scholar Academy. We have a relationship between our university and, for example, Peking University, Tsinghua University, Fudan University, and Chinese Agriculture University. We exchange students and scholars and we develop research projects in that way.

There are brilliant people everywhere. People can make tremendous contributions regardless of their circumstance, whether they are uneducated farmers on a hill in Thailand or top scientists in Boston. I’ve worked a lot in Thailand. My colleagues there often have unique perspectives based on their experiences and have made tremendous contributions. Our goal is to have a global community of science and to have those individuals contributing their perspectives, knowledge, and brilliance. This is especially important because many developing countries face unique challenges. The best way to address them is to have their citizens trained in the West then go back to meet the challenges at home. I see it as part of the research enterprise. It's humanity.

Related to the diversity of science is women's participation. It's a huge issue globally—even in countries that have a long-standing tradition of women in science. In Iran, for instance, there are many women scientists. In the USA, we have a lot of women biologists; fewer work in physical science. And there are countries where women have not traditionally gone into science. It's highly variable. If we are going to solve the problem, if we are going to move science forward to benefit humanity, we will need a lot of people doing that, not just half of the population.

We need an environment where the lessons of science can be taught without the fear of censure or even in some cases reprisals.

—Barbara Schaal

**NSR:** What have you been doing in China?

**Moran:** We’ve been working closely with China in the last decade. What's really important is to understand China—culturally, scientifically and politically. Our work in China is more about what Chinese researchers need and how we can help because we believe in science without borders. It's mostly on the career side, such as writing skills, presentation skills, and career plans. For instance, we run career workshops at least four times a year, where our editors talk about how to publish in *Science* and other *Science* family publications. This helps Chinese researchers better understand the peer review process, among other publication aspects. It's been very effective.

We send editors to attend conferences in China and to pay regular visits to top Chinese universities and research institutes. They meet principle investigators and discuss their research. This helps to raise awareness for both sides. This process reflects our desire at *Science* to listen to the needs of Chinese researchers, to understand how we can work together. On the AAAS side, we have, for example, been in discussion with the Chinese Association of Science and Technology about running a workshop on research ethics. They developed such a workshop, which was well received. It's really important for us to listen to the needs of Chinese researchers and then work with them to develop useful programs.

**NSR:** What are the main challenges in fostering this relationship with China?

**Schaal:** My feeling is that given the status of *Science* publications, there is no need for encouragement. *Science* is, of course, an extraordinary journal and the open access journal *Science Advances* is also very exciting, an amazing experiment that has really filled the need. As in the case with other journals such as *Nature* and *Cell*, the challenges are that it's as much about superb science as it is about the art of preparing for a submission. It's no mean feat for scientists from a country where English is not the native language. It's something quite heartbreaking for many of us when we know the science is superb, but when there are ten articles for one space, not all superb science can get in.

**NSR:** What are the submission and acceptance rates of papers from China?

**Moran:** Since 2011, submission from China has increased by 15%. More importantly, the quality of those submissions is tremendous. The rate of acceptance has increased by 30% since 2011. What's really interesting is that the Thousand Talents Program [developed to attract highly accomplished scientists abroad back to China] has really helped in the past decade. You can see it. Now some of the Western-trained researchers have gone back to China. They have had a critical role in boosting Chinese science and are training the next generation.

**Schaal:** Another aspect is that science is highly international these days. There are many Chinese students and post-docs overseas. There are a lot of exchanges and collaborations between Chinese and foreign laboratories. This reflects in the publications from China, which are often very international. That has really made a big difference. It's not just the Chinese science *per se* but also the globalization of science.

**NSR:** What are the weaknesses of Chinese science and Chinese authors trying to publish in the *Science* family publications?

**Schaal:** I think language is definitely a challenge. Telling the story of a scientific research project in a clear, succinct, and engaging way is challenging for native English speakers. It's doubly challenging for those whose mother tongue is not English. For papers in journals that publish a limited number of papers per issue, there has to be a point. The whole paper has to be written towards that point logically. And you have room to make just one or two points. I know that given the high technologies it takes to do science nowadays, it's very easy—certainly for me—to get distracted by the methodology. You get so distracted instead of going to the end conclusion; you meander around because you are talking about the methodology. This just won’t do. Most *Science* papers are crisp and clean.

**Moran:** I totally agree. It's hard enough when English is your first language. That's why we offer technical-writing workshops to help. They are given by editors visiting Chinese universities and research institutes. We also try to develop relationships, such as at CAS (Chinese Academy of Sciences), CAMS (Chinese Academy of Medical Science), and CAST (Chinese Association for Science and Technology). It's really important to develop those relationships. When we do that with our editors, we can learn first-hand what the researchers’ concerns are and how we can help.

**NSR:** What is the motivation or vision or rationale behind this kind of proactive collaboration?

**Schaal:** To improve science. That's what we believe in deeply and it underlies the vision of AAAS. Science is not a political construct. It's not a national construct. It's a global construct for the betterment of humankind.

**Moran:** To expand on that, we offer prizes. We are offering a prize with PINS Medical and having discussions around potential prizes with leading research institutes and enterprises. They are doing pioneering research in neuromodulation and have collaborated notably with the US National Institutes of Health. The results they are getting are fantastic. They will help treat depression and Parkinson's. This is what we want to do: to improve science and help humankind in that way.

**NSR:** How would you evaluate the global enterprise of science right now?

**Schaal:** In the past decade or so, there has been a concern in the USA and around the world that the position of science in the world is eroding. We see cases in which the role of science is marginalized and that science is discounted as just another belief system or another political system. We need to be aware of the intensifying hostility towards science in many parts of the globe. The scientific community needs to have a clear voice. We need to make the case for science. Science is a global public good. It's central to the functioning of a government, to the well-being of its citizens, and to the health of our economy, and the health of our planet.

We need scientific advice for the development of policies across a wide range of government activities. Policies based on

Our work in China is more about what Chinese researchers need and how we can help because we believe in science without borders.

—Bill Moran

proper scientific work produce positive outcomes: they keep people safe, improve the quality of drinking water, improve access to education and jobs, and protect our planet. This is science for policy, science for good policy. The role of science is to speak the truth to power and to give an unbiased assessment of the current situation and the state of knowledge of a particular area. Policies need to have a basis that reflects fact and evidence.

In order for nations to reap these benefits, science needs to flourish and the basic components of the process need to be protected. This includes open communication, not policies that stifle communication. This includes global science, not policies that suppress the movement of individuals and ideas. To address challenges such as climate change, new infectious diseases, and new sources of clean energy, we need the global community to work on them. These are not political issues. They transcend political and national boundaries.

It's our obligation as scientists and citizens, we need to speak up for science, we need to make the case for science. Be a force for science.

